# Optimal control on a mathematical model to pattern the progression of coronavirus disease 2019 (COVID-19) in Indonesia

**DOI:** 10.1186/s41256-020-00163-2

**Published:** 2020-08-05

**Authors:** Novi Reandy Sasmita, Muhammad Ikhwan, Suyanto Suyanto, Virasakdi Chongsuvivatwong

**Affiliations:** 1 Department of Statistics, Faculty of Mathematics and Natural Sciences, Universitas Syiah Kuala, Banda Aceh, 23111 Indonesia; 2 Graduate School of Mathematics and Applied Sciences, Universitas Syiah Kuala, Banda Aceh, 23111 Indonesia; 3 Department of Public Health and Community Medicine, Faculty of Medicine, Universitas Riau, Pekanbaru, 28000 Indonesia; 4 Epidemiology Unit, Faculty of Medicine, Prince of Songkla University, Hat Yai, 90110 Thailand

**Keywords:** COVID-19, Optimal control, Mathematical model, Indonesia

## Abstract

**Background:**

Understanding the pattern of COVID-19 infection progression is critical for health policymakers. Reaching the exponential peak of cases, flattening the curve, and treating all of the active cases are the keys to success in reducing outbreak transmission. The objective of this study was to determine the most effective model for predicting the peak of COVID-19 in Indonesia, using a deterministic model.

**Methods:**

The SEI2RS model considers five strategies for control, namely: large-scale social restriction (***u***_**1**_), contact tracing (***u***_**2**_), mass testing (***u***_**3**_)***,*** case detection and treatment (***u***_**4**_), and the wearing of face masks (***u***_**5**_)**.** Three scenarios were developed, each differentiated by the controls. The model used April 10, 2020, and December 31, 2020, as the initial and final times.

**Results:**

The simulation results indicated that the peak of COVID-19 cases for scenarios 1, 2, and 3 occur on the 59th day with 33,151 cases, on the 38th day with 37,908 cases, and on the 40th day with 39,305 cases. For all of the scenarios, the decline phase shows a slow downward slope and about 8000 cases of COVID-19 still active by the end of 2020.

**Conclusion:**

The study concludes that scenario 2, which consists of large-scale social restriction (*u*_1_), contact tracing (*u*_2_), case detection and treatment (*u*_4_), and the wearing of face masks (*u*_5_), is the most rational scenario to control COVID-19 spreading in Indonesia.

## Background

**Fig. 1:**
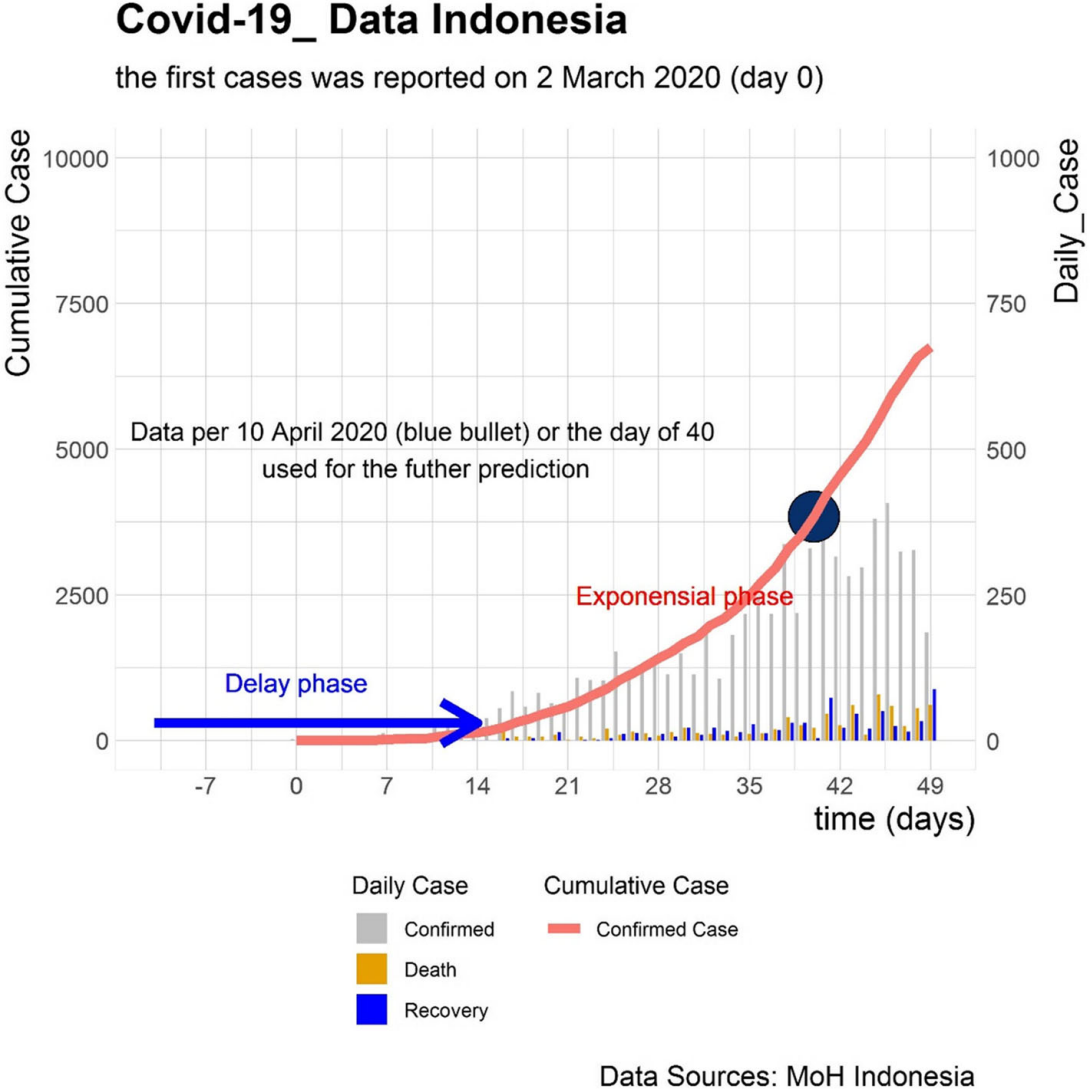
COVID19 Situation in Indonesia

The outbreak of coronavirus disease (COVID-19) has spread to nearly every country around the world. With a high density population, Indonesia is predicted to have a high number of infectious persons and, consequentially, suffer over a longer time period [1]. The first positive cases of COVID-19 in Indonesia were confirmed with two cases in Java Island on March 2, 2020, then spreading to other islands [2, 3]. As of the first week of April, there are about 3000 cases reported (Fig. 1). However, there is skepticism about whether the true number of COVID-19 cases in the community is higher than reported, due to inadequate testing and under detecting [4].

Without well prepared interventions, the number of COVID-19 cases will grow exponentially. Though delayed, the Indonesian government has taken numerous measures to control the community spread of COVID-19. Control measures, such as large-scale social restriction policies in specific provinces, a campaign for frequent hand washing and face mask use, and rapid testing suspected cases, have been implemented across Indonesia [5]. Indonesia benefits from being a country of a thousand islands, as this geography may delay disease transmission from the main epicenter on Java Island to surrounding islands.

Considering limited knowledge of COVID-19 in Indonesia, where the number of cases has been fast-growing since the onset of the epidemic, this study proposes a deterministic mathematical model based on the susceptible (*S*), exposed (*E*), infectious (*I*), recovered (*R*)-(SEIR) model [6–8]. Selected control variables were simulated, representing measures that have been and will be implemented by the Indonesian government to detect and reduce COVID-19 transmission. These measures include large-scale social restriction (*u*_1_), contact tracing (*u*_2_), mass rapid antibody testing (*u*_3_), case detection and treatment (*u*_4_), and the wearing of face masks (*u*_5_). Using a mathematical model as an approach to solving problems can help in explaining current phenomena [9, 10]. A mathematical model may help us to understand patterns in disease outbreak, especially that of COVID-19, and lead to a more public health informed policy making process [11, 12].

Applying optimal control to a mathematical model can predict, forecast, estimate, or choose the best scenario to eliminate a disease in a dynamical system, based on epidemiological characteristics [13]. In the phenomenon of the COVID-19 outbreak, there are lags among the real on-going spread of infection, the case detection and report and the response action. If the case load could be properly predicted, the government would have the ability to fine tune their reaction, such as adjustment of the intensity of social distancing, preparation of medical resources to cope with the case load, and planning for socio-economic recovery at proper time points.

## Methods

### SEI2RS model formulation

A deterministic mathematical model is applied by dividing populations into several compartments. The basis of the mathematical model in this study, SEIR, is modified to be the susceptible (*S*), exposed (*E*), carrier (*I*_1_), infectious (*I*_2_), recovery (*R*), susceptible (*S*)-(SEI2RS) Model. The cumulative number of cases (in the infectious compartment (*I*_2_)) will increase depending on the movement of the suspect population (in the exposed compartment (*E*)) and the number of undetected cases (in the carrier compartment (*I*_1_)). The number of *I*_2_ cases will decrease as there is increased movement of infectious cases to the recovery compartment. This compartmental model was modified not only to obtain a realistic mathematical model for the Indonesian setting, but to also follow the pathogenesis of COVID-19.

Susceptible (*S*) refers to the individuals deemed susceptible to COVID-19 infection during the period of outbreak. A susceptible person may be a healthy individual, but based on currently available information and clinical expertise, are at higher risk for severe illness, such as older adults and people of any age who have serious underlying medical conditions. Since local transmission has been reported in Indonesia, all patients presenting with symptoms of acute respiratory infection in primary care or the accident and emergency department of a hospital (first contact with the healthcare system) will be considered as suspected cases, regardless of their presumed susceptibility.

Indonesian guidelines divide the patient’s health status by confirmed cases and suspect cases. Exposed (*E*) individuals refer to suspect cases. Suspected cases include Person Under Monitoring (PUM) and Patient Under Supervision (PUS). PUM is an individual who traveled to the coronavirus epicenter or has made contact with a person suspected to be positive for COVID-19 but does not show any symptoms themselves. PUS is an individual who has shown symptoms of coronavirus infection, ranging from mild to severe pneumonia. A PUS is then categorized as a positive COVID-19 case if a swab test produces positive results.

Also included in the model, the carrier (*I*_1_) refers to individuals in the community that go undetected and may spread the disease while unaware of their infectious status. This category contains people who remain asymptomatic or develop major noticeable symptoms but are not detected as a suspected case in health facilities. This study considers carrier (*I*_1_) as a compartment because the previous study found around 86% of those infected had remained asymptomatic (or had flu-like mild symptoms). This group of individuals makes transmission control particularly difficult, as they may unknowingly transmit the disease to vulnerable individuals (such as elderly people and people with underlying diseases) [14].

Infectious (*I*_2_) individuals refer to confirmed COVID-19 cases that are detected by a health facility and then receive treatment. Recovery (*R*) individuals refer to the people who recover from COVID-19 infection due to their improving immunity and successful hospital treatment. The total population considered at the time (*t*) in this study can be denoted by N (t), as calculated in the following equation:

1 $$ N\ (t)=S(t)+E(t)+{I}_1(t)+{I}_2(t)+R(t) $$

Parameters were also developed while implementing this mathematical model. *Λ* symbolizes the natural birth rate, (*μ*) symbolizes the natural death rate, including the susceptible, exposed, carrier and recovery compartments, and (*μ*_1_) is the death rate in the infectious compartment. There are three infected rates used in the model; the infected rate of *S* to *I*_1_ (*α*_1_), the infected rate of *S* to *I*_2_ (*α*_2_), and the infected rate of *S* to *E* (*α*_3_). *β*_1_ is the diagnostic error rate of *E* to *S* and *β*_2_ is the development rate of *E* to *I*_2_. *γ*_1_ and *γ*_2_ are the recovery rates from *I*_2_ to *R* explained by immunity, and the development rate of *I*_1_ to *I*_2_ because of the positive rapid test result, respectively.

Furthermore, *θ*_1_ and *θ*_2_ are the treatment success rates from *I*_2_ to *R*, and the diagnostic error rate of *I*_2_ to *S*. *θ*_2_ is very likely to occur in developing countries because of limited access to resources and advanced clinical capabilities. This model considers varying possible results for the specified rates. The last parameter in this model is the loss of immunity rate of *R* to *S*. Assumptions for this model include waning immunity, in that someone who has recovered from COVID-19 will later lose their immunity and return to susceptible individual category.

**Fig. 2:**
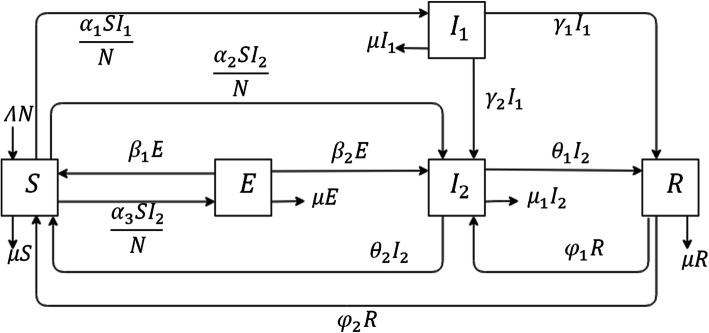
Flowchart of COVID-19 Transmission in Indonesia

All parameters included in the mathematical model are assumed to be positive and constant. Based on these assumptions, a flowchart for COVID-19 transmission could be constructed, as shown in Fig. 2. According to the flowchart, the differential equations used for the model are:

2 $$ {\displaystyle \begin{array}{l}\frac{dS}{dt}=\varLambda \mathrm{N}+{\varphi}_2R+{\beta}_1E+{\theta}_2{I}_2-\frac{\alpha_1S{I}_1}{N}-\frac{\alpha_2S{I}_2}{N}-\frac{\alpha_3S{I}_2}{N}-\mu S\\ {}\frac{dE}{dt}=\frac{\alpha_3S{I}_2}{N}-{\beta}_1E-{\beta}_2E-\mu E\\ {}\frac{dI_1}{dt}=\frac{\alpha_1S{I}_1}{N}-{\gamma}_1{I}_1-{\gamma}_2{I}_1-{\mu I}_1\\ {}\frac{dI_2}{dt}=\frac{\alpha_2S{I}_2}{N}+{\beta}_2E+{\gamma}_2{I}_1+{\varphi}_1R-{\theta}_1{I}_2-{\theta}_2{I}_2-{\mu}_1{I}_2\\ {}\frac{dR}{dt}={\gamma}_1{I}_1+{\theta}_1{I}_2-{\varphi}_1R-{\varphi}_2R-\mu R\end{array}} $$

The parameters used in Eq. (2) are constant and non-negative, detailed in Table 2. Then, Eq. (2) has a biological region as a solution 5-dimensional positive real numbers denoted as: [23]

3 $$ \Gamma =\left\{\left(S,E,{I}_1,{I}_2,R\right)\in {\mathbb{R}}_{+}^5:0\le N=S+E+{I}_1+{I}_2+R\le \frac{\varLambda N}{\mu}\right\} $$

### Endemic equilibrium and its stability

The equilibrium point is obtained by accounting for the first derivative of the equation [24]. This derivative describes the gradient of a particular value environment, which in this case, is the peak point or valley. A problem in initial values and parameters applied to the system model Eq. (2) results in instability at the equilibrium point (*S*^∗^ = 895,722,037, *E*^∗^ = 0, ${I}_1^{\ast }$ = 0, ${I}_2^{\ast }$ = 459,601 and *R*^∗^ = 2071). The eigenvalue analysis of the *J* matrix is in the form of five negative values, which guarantees that the resulting system is asymptotic and stable. The eigenvalues obtained are − 0.0255, − 0.0346, − 1.9516 × 10^− 5^, − 0.0033 and − 0.0029.

### Optimal control

The control variables are strategies to detect and reduce COVID-19 transmission in Indonesia, including large-scale social restriction (*u*_1_), contact tracing (*u*_2_), mass testing (*u*_3_), case detection and treatment (*u*_4_) and the wearing of face masks (*u*_5_), as proposed in currently evolving scenarios. The large-scale social restriction is an effort to slow the spread of coronavirus by encouraging people to do their routine activity at home, keep a minimum of 1.5 m distance from other individuals while talking, and avoid public gatherings. Contact tracing is a systematic effort to track and trace people who may have been exposed to an infectious disease, COVID-19 in this instance, to categorize them as a Person Under Monitoring (PUM) or a Patient Under Supervision (PUS). If available, the rapid test would be conducted on the individual to confirm their infection status.

Mass testing is an effort to aggressively test healthy individuals who remain asymptomatic. Mass testing will detect new infections earlier and provide a more complete picture of spread within a community, by capturing positive cases that would have never shown symptoms. This testing approach could be implemented by using the rapid test. Case detection and treatment is an effort to detect the virus in exposed individuals, so they can be moved to the infectious individual category using polymerase chain reaction (PCR) testing methods and be guaranteed high-quality treatment. The last control is the widespread wearing of face masks. This control is used to protect an individual not currently infected by COVID-19 and to control the transmission from an asymptomatic virus carrier.

Based on the function of these controls, three scenarios were made to estimate the incidence and the peak of cumulative COVID-19 cases in Indonesia. The first scenario includes three control variables, large-scale social restriction (*u*_1_), case detection and treatment (*u*_4_), and wearing of face masks (*u*_5_). The second scenario includes large-scale social restriction (*u*_1_), contact tracing (*u*_2_), case detection and treatment (*u*_4_), and the wearing of face masks (*u*_5_). The third scenario applies all of the previously listed control functions.

In the model, large-scale social restriction (*u*_1_) and mass testing (*u*_3_) are included in compartment *S* and *I*_1_. Employing large-scale social restriction (*u*_1_) on compartment *S* will reduce the number of people infected with COVID-19 in the subsequent time period. The mass testing (*u*_3_) aims to move a number of susceptible individuals to the more accurate infectious individuals category. At the same time, contact tracing (*u*_2_) is applied to compartment *E.* This contact tracing approach aims to move exposed individuals to infectious individuals in the compartment *I*_1_.

Furthermore, the case detection and treatment (*u*_4_) control function is employed on compartment *I*_1_. This process aims to reduce the number of infectious individuals by moving them to the recovered individuals category. The last control strategy is to wear face masks (*u*_5_). Face masks as a mitigation strategy is put on compartment *S*, *E*, and *I*_1_. The aim of (*u*_5_) is to keep the individuals in their specified compartment, as well as prevent others from transferring between compartments, during the outbreak of COVID-19.

Based on the optimal control problem, the formulated mathematical model is:

4 $$ {\displaystyle \begin{array}{l}\frac{dS}{dt}=\varLambda \mathrm{N}+{\varphi}_2R+\left(1+{u}_2-{u}_5\right){\beta}_1E+\left(1+{u}_4\right){\theta}_2{I}_2-\left(1-{u}_1-{u}_5\right)\frac{\alpha_1S{I}_1}{N}-\left(1-{u}_1+{u}_3-{u}_5\right)\frac{\alpha_2S{I}_2}{N}-\left(1-{u}_1-{u}_5\right)\frac{\alpha_3S{I}_2}{N}-\mu S\\ {}\frac{dE}{dt}=\left(1-{u}_1-{u}_5\right)\frac{\alpha_3S{I}_2}{N}-\left(1+{u}_2-{u}_5\right){\beta}_1E-\left(1+{u}_2-{u}_5\right){\beta}_2E-\mu E\\ {}\frac{dI_1}{dt}=\left(1-{u}_1-{u}_5\right)\frac{\alpha_1S{I}_1}{N}-\left(1+{u}_1-{u}_5\right){\gamma}_1{I}_1-\left(1-{u}_1+{u}_3-{u}_5\right){\gamma}_2{I}_1-{\mu I}_1\\ {}\frac{dI_2}{dt}=\left(1-{u}_1+{\mathrm{u}}_3-{u}_5\right)\frac{\alpha_2S{I}_2}{N}+\left(1+{u}_2-{u}_5\right){\beta}_2E+\left(1-{u}_1+{u}_3-{u}_5\right){\gamma}_2{I}_1+{\varphi}_1R-\left(1+{u}_4\right){\theta}_1{I}_2-\left(1+{u}_4\right){\theta}_2{I}_2-{\mu}_1{I}_2\\ {}\frac{dR}{dt}=\left(1+{u}_1-{u}_5\right){\gamma}_1{I}_1+\left(1+{u}_4\right){\theta}_1{I}_2-{\varphi}_1R-{\varphi}_2R-\mu R\end{array}} $$

The goal of the control variables is to find the optimal scenario to minimize the exposed, carrier, and infectious individuals. To reduce COVID-19 transmission, infectious individuals need to be treated while simultaneous efforts are made to screen and treat exposed and carrier individuals to prevent the occurrence of new COVID-19 cases. The efforts made in the objective function are assumed to be a non-linear and quadratic function. The quadratic function is the common form of an objective function in an OCT problem [25]. The objective function minimized in this model is as follows:

5 $$ J\left({u}_1,{u}_2,{u}_3,{u}_4,{u}_5\right)={\int}_{t_0}^{t_f}\left(E+{I}_1+{I}_2+\frac{1}{2}{A}_1{u}_1^2+\frac{1}{2}{A}_2{u}_2^2+\frac{1}{2}{A}_3{u}_3^2+\frac{1}{2}{A}_3{u}_4^2+\frac{1}{2}{A}_3{u}_4^2\right) dt $$

The model parameters *t*_0_ and *t*_*f*_ are the initial and final time. They are taken as *t*_0_= April 10, 2020, and *t*_*f*_= December 31, 2020, for a duration of 266 days. The weight coefficients, *A*_1_*, A*_2_, *A*_3_, *A*_4_, and *A*_5_ are chosen to balance the population value during the minimization process [25]. The study aims to obtain the optimal control while satisfying ${u}_1^{\ast },{u}_2^{\ast },{u}_3^{\ast },{u}_4^{\ast }$ and ${u}_5^{\ast }$.

6 $$ J\ \left({u}_1^{\ast },{u}_2^{\ast },{u}_3^{\ast },{u}_4^{\ast },{u}_5^{\ast}\right)={\displaystyle \begin{array}{c}\mathit{\min}\\ {}\Omega \end{array}}\ J\left({u}_1,{u}_2,{u}_3,{u}_4,{u}_5\right) $$

where Ω = {(*u*_1_, *u*_2_, *u*_3_, *u*_4_, *u*_5_ )*ϵ L*^1^ (*t*_0_; *t*_*f*_)| 0 ≤ *u*_*i*_ ≤ 1 for *i* = 1, 2, 3, 4, 5}. In the *u*_*i*_ control range, no investment in control is made if the value of *u*_*i*_ = 0. In When the control value is 1, the maximum effort is made on the control in question. The vector Ω is considered to be in the Lebesgue space with *p*-norm, which is *p* = 1. The vector was stated as Lebesgue measurable and was successful in several cases of optimal control such as malaria, tuberculosis, and tumor treatment [23, 26–28]. Euclid space, *L*^2^(*t*_0_, *t*_*f*_), was considered and the functional analysis results were shown in Appendix 2.

The control strategy was assumed to affect the model in all scenarios, so several cases that accommodate these assumptions were considered. The weight parameters in the model were then set as *A*_1_ =25, *A*_2_ =15, *A*_3_ =10, *A*_4_ =90, and *A*_5_ =20. Values of *A*_*i*_ were weighted so that the control variable *u*_*i*_ could be considered as the minimum objective. Further, the Fourth order Runge-Kutta method was also considered to solve the optimal control problem (power of error about $\mathcal{O}\left({h}^5\right),h<1$). Analyses were performed using MATLAB software and visualization of the results was done on R software.

### State variables and parameters

For computation, the newest COVID-19 data from Indonesia is used as baseline data. Baseline refers to data per April 10, 2020, or 40 days after the first reported cases. Appropriate estimations are provided by looking at the stability of the data pattern while considering the results of current national policies being implemented, such as large-scale social restriction, mass rapid antibody testing, and obligatory face mask policy.

The study was conducted by gathering the initial values of state variables and parameters from various valid sources (Table 1). The number of exposed individuals (*E)*, reported infectious cases (*I*_1_), and recovery *(R)* cases was obtained from provincial and national data sources. The number of viral carriers (*I*_2_) was best estimated based on other studies [14]. The number of susceptible individuals (*S*) was estimated by calculating the total population minus the number of exposed individuals, carrier individuals, infectious individuals, and recovery individuals, as follows: *S* = *N*- *E*- *I*_1_- *I*_2_- *R*.

**Table 1:** Initial value of state variable for COVID-19 transmission in Indonesia

Sy	Value	Reference	Sy	Value	Reference
*S*	267,823,870	Data fitted	*I*_2_	3842	The Government of the Republic of Indonesia [17]
*E*	148,401	Data fitted	*R*	286	The Government of the Republic of Indonesia [17]
*I*_1_	23,601	Data fitted	*N*	268,000,000	World Health Organization [29]

*Abbreviation*: *Sy* Symbol, The unit of state is the number of individuals

The parameter values used in the study were chosen and calculated to be as close to Indonesia’s true condition as possible, see Table 2. Some parameters in the model were calculated through estimation because precise data were not available. According to the pathogenesis of COVID-19, infectious individuals can infect up to 2–2.5 other people in the course of 4 days, based on the reproduction number of COVID-19 cases [30, 31]. Based on this statement, the infection rate between susceptible individuals and exposed individuals was calculated by the number of infectious individuals multiplied by 2.5 and then divided by the number of susceptible individuals, as shown by: *α*_3_ = (*I*_2_ × 2.5)/ *S*. Following the same method, the infection rate between susceptible individuals and infectious individuals is also calculated: *α*_1_ = (*I*_1_ × 2.5)/ *S*. Additionally, the loss immunity rate (*φ*_2_) is estimated based on the calculation of 1 minus the death rate in the *R* compartment and relapse rate, as follows: *φ*_2_ = 1 − *μR* − φ_2_. Based on these formulae, the resulting values are *α*_3_ = 3.586 × 10^− 5^, *α*_1_ = 2.203 × 10^− 4^ and *φ*_2_ = 0.96228. The diagnostic error rate of *E* to *S* is assumed to be 95% until December 2020. *θ*_2_ is the diagnostic error rate of *I*_2_ to *S*. The diagnostic error rate is the rate of false positive results of a rapid test, which although rare, still occur.

**Table 2:** Value of parameter for COVID-19 transmission in Indonesia

Symbol	Value	Reference
*Λ*	0,01888	World Bank [15]
*μ*	0.00712	World Bank [16]
*μ*_*t*1_	0.087	The Government of the Republic of Indonesia [17]
*α*_1_	2.203 × 10^− 4^	Data fitted
*α*_2_	0.036	The Goverment of West Java, Indonesia [18]
*α*_3_	3.586 × 10^− 5^	Data fitted
*β*_1_	0.950	Assumed
*β*_2_	0.4	Qifang et al [19], Prem et al [20]
*γ*_1_	0.075	Ivorra et al [21]
*γ*_2_	0.036	The Goverment of West Java, Indonesia [18]
*θ*_1_	0.086	The Government of the Republic of Indonesia [17]
*θ*_2_	0.005	Assumed
*φ*_1_	0.03	Fidinillah [22]
*φ*_2_	0.9629	Data fitted

The unit of state is the number of individuals

## Results

Three scenarios were simulated by plotting all control strategies as a function of time in the model. Scenario 1 includes large-scale social restriction (*u*_1_), case detection and treatment (*u*_4_), and the wearing of face masks (*u*_5_). Scenario 2 includes large-scale social restriction (*u*_1_), contact tracing (*u*_2_), case detection and treatment (*u*_4_), and the wearing of face masks (*u*_5_). Lastly, scenario 3 added control mass testing (*u*_3_) to all controls included in scenario 2.

**Fig. 3:**
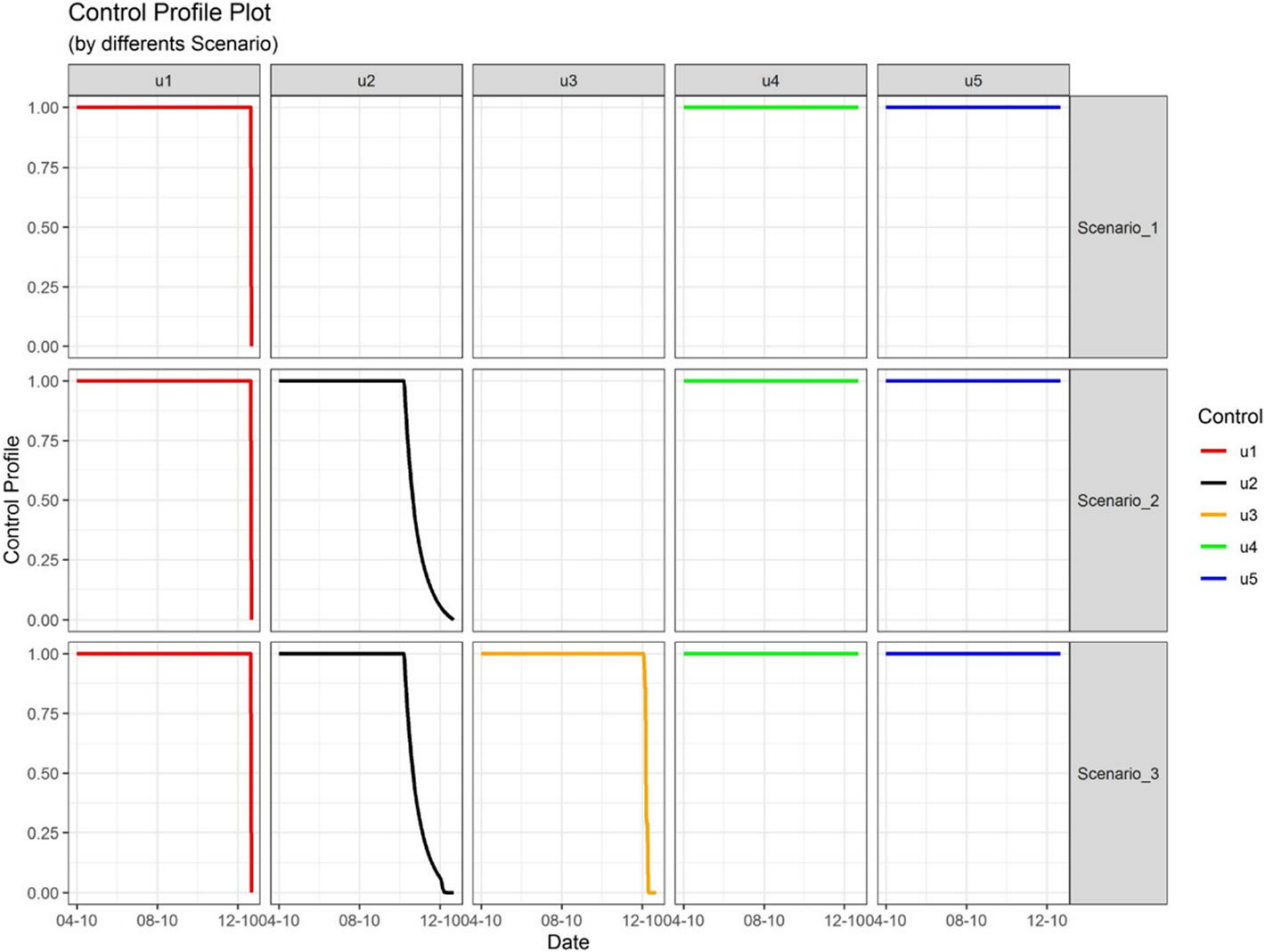
Control Profile of COVID-19 Cases on Different Scenario in Indonesia

In all of the modeled scenarios, the optimal control *u*_1_ remained near the upper bound, around 1.00, until 264 days after the initial application. After that critical point, *u*_1_ dropped dramatically to the lower bound at the final captured time frame. The optimal control *u*_4_ and *u*_5_ for scenario 1, 2, and 3 were continuously at the upper bound from the initial time until the final time recorded. *u*_2_ and *u*_3_ react differently in the control profile, as *u*_2_ was fully applied for only a few months and remained around 0.99 for scenario 2 and 3, before decreasing linearly towards the lower bound. The last, *u*_3_ was around the upper bound at 0.99 until 246 days after initiation, and then it decreased linearly towards the lower bound by the final time recorded (Fig. 3).

**Fig. 4:**
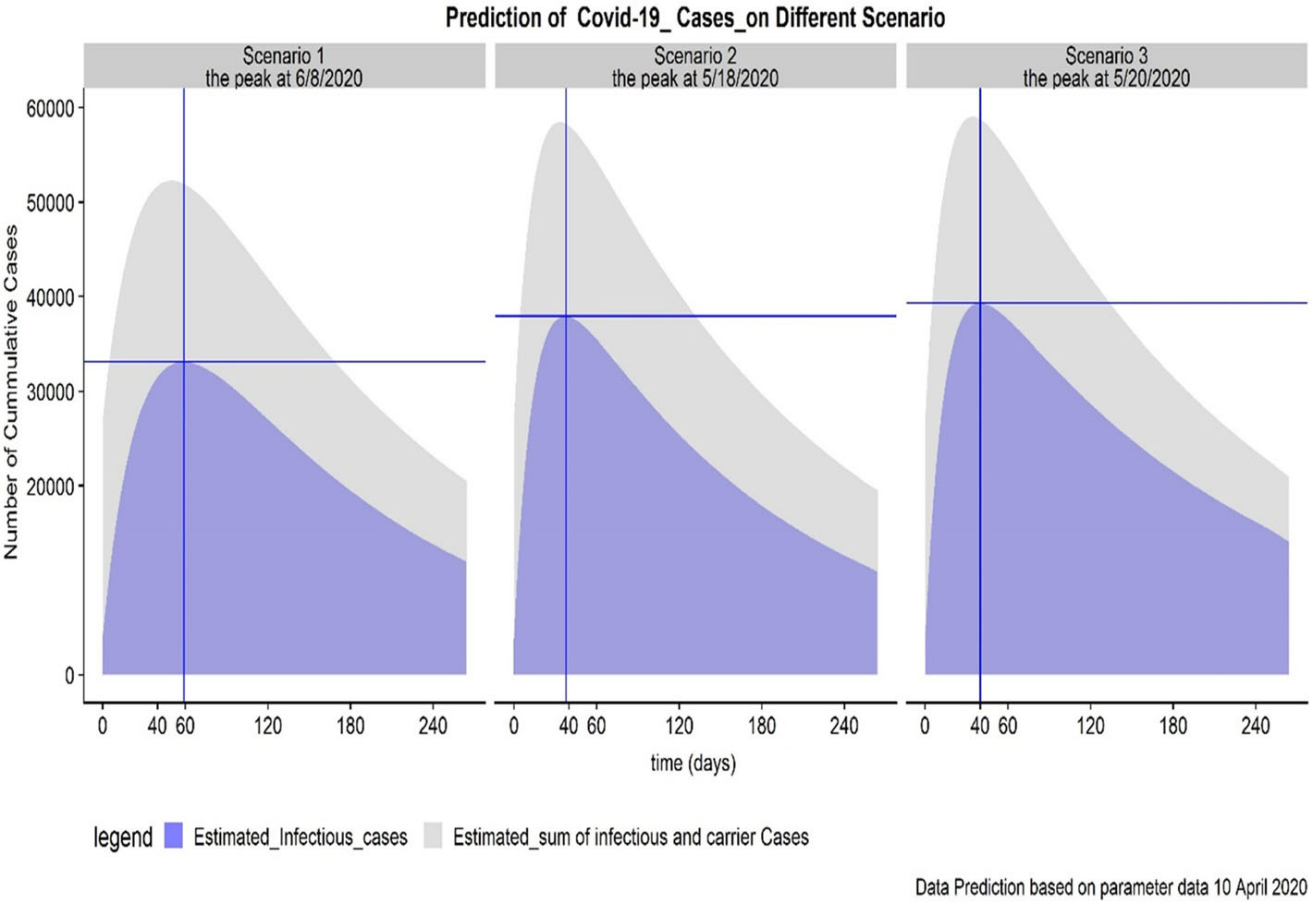
Prediction of COVID-19 Cases on Different Scenario in Indonesia

The changing of each control profile is implied in different scenarios and resulted in a varying amount of change between the population of each compartment. As shown in Fig. 4, each scenario will have a different impact on the spread of COVID-19 and the culmination of cases. The objective function of the proposed mathematical model, applying optimal control as in Eq. (5) by Eq. (6), is to minimize the number of suspect (or exposed) individuals, the number of carrier individuals (or undetected cases), and the number of infectious individuals, in Indonesia.

The simulation showed that the number of suspect individuals, the number of carrier individuals, and the number of infectious individuals at final time for all scenarios is 148,401 cases, 23,601 cases, and 3842 cases, respectively. Figure 4 shows the peak in the number of infectious individuals for each of the simulated scenarios. The peak for scenario 1, 2, and 3 are 33,151 cases on the 59th day, 37,908 cases on the 38th day, and 39,305 cases on the 40th day. Predictions extend until the end of 2020, showing that cases of COVID-19 will still be active.

To examine the impact of the control profile on overall transmission, the number of suspect individuals, the number of carrier individuals, and the number of infectious individuals was counted at the end of projected time for each scenario. The analysis shows the projected counts of 15 cases, 8473 cases, and 11,935 cases, respectively, in Scenario 1. At the final time reported in scenario 2 there are 0 cases, 8473 cases, and 10,919 cases for the number of suspect individuals (or exposed), carrier individuals (or undetected cases), and infectious individuals, respectively. In scenario 3, the number for suspect individuals, carrier individuals, and infectious individuals are 0 cases, 6802 cases, 14,016 cases, respectively. Based on the results, scenario 2 shows the shortest amount of time between control measure initiation and the peak of COVID-19 cases in Indonesia. The peak of COVID-19 cases for scenario 2 and 3 does not differ significantly. However, the duration of time to the peak of infectious cases will take the most time in Scenario 1, an estimated 59 days.

## Discussion

This paper highlights the changes to COVID-19 control measure variables to estimate the time to the peak of cases, while attempting to limit the spread of the outbreak in several scenarios. Analysis is based on the Susceptible-Exposed-Infectious-Recovered’ (SEIR) model, which has been globally accepted. A main limitation of this model when considering application to other countries is the variance in control strategies implemented by the region’s government, which may reflect a level of decentralization of power in COVID-19 control. Scenario 2 is the fastest projected scenario to reach the peak of COVID-19 cases in Indonesia. This scenario includes the implementation of large-scale social restriction (*u*_1_), contact tracing (*u*_2_), case detection and treatment (*u*_4_), and the wearing of face masks (*u*_5_).

The number of detected cases was accounted for, as this count is highly correlated with the number of tests available and conducted. Compared to other countries, Indonesia has a relatively small number of tests being conducted in health facilities and throughout the community. Indonesia has conducted just 0.13 COVID-19 tests per thousand people, or 34,975 in total (as of April 16, 2020) [32]. There are still many people who have not been confirmed as COVID-19 patients and may be unknowingly spreading the virus through the community, due to the limited resources and government capability to conduct tests at an adequate rate. As of the first week of April, the capacity for daily PCR testing is still limited to just 12 laboratories throughout Indonesia. When this study began, the Indonesian government had attained a testing machine that can process up to 1000 tests a day, and planned to place it in more than 49 laboratories through-out the country. In this rapidly evolving global crisis, our prediction of the peak of the epidemic should be interpreted based on parameters per April 10, 2020.

Theoretically, when the government and healthcare facilities have the ability to test for all cases in the community and treat the detected infectious cases, the number of new COVID-19 cases will slow until completely stopped. Results show that mass testing (*u*_3_) does not have a significant effect on the scenario’s timeline or peak. This conclusion may be attributed to the antibody rapid test assumption in this model, which failed to increase the number of cases detected. An increase in availability and implementation of PCR tests is suggested. Scenario 2 suggests that it is important to detect at least 40,000 cases in the first week of May 2020 and to identify efforts to reach the carrier cases in the simulation. Resulting scenarios highlight the key to successful outbreak control is to find the infectious case as fast as possible, so the patient can be treated as well as socially restricted, separating the carrier cases from other individuals that may be susceptible to the disease.

Learning from other country’s actions, it is also possible that Indonesia will experience a second wave of COVID-19, resulting in a second peak. The influx of possibly infectious people traveling from other countries or from delayed disease onset are risks that need to be considered. Though not considered in the model, the possibility of recovering cases being re-infected, or experiencing a relapse needs to be examined. Large-scale social restriction (*u*_1_) should be maintained, as well as wearing face masks (*u*_5_) and case detection and treatment programs (*u*_4_). The decline in number of infectious cases looks slow sloping and shows that even at the end of 2020, there will still be about 20,000 cases. This result can be explained by a relatively slow recovery rate and a simultaneous movement of individuals from the susceptible to the infectious compartment. The discovery of effective drugs as treatment may reduce the number of infectious cases at a rapid rate, unseen up to the current time. A vaccine is the most effective solution for preventing movement from the susceptible group to active infectious cases, thus reducing the spread of the disease. These assumptions point to COVID-19 becoming endemic in parts of the world, much like a number of other infectious diseases.

Assumptions for this model were developed from reported cases that had been tested prior to 10 April 2020. During this time, there may have been significant change in policy or unsuccessful policy implementation, deeming the prediction inaccurate. Indonesia can consider implementing the scenario 2 control profile, with limited resources, throughout the year 2020, based on this study. This scenario requires increased public awareness to adhere to physical distancing and face mask guidelines. The government can continue their efforts to accurately track and trace cases, as well as improving the health care facilities. Due to non-significant results, mass antibody testing is not recommended. Further evaluation research would be useful to measure the results of this policy.

This model is relatively simple and estimates are concurrent with previous studies. The model holds merit in its consideration of essential elements, including the control variable from government efforts, although geographical compartment groups that may impact the number of cases in each scenario are not considered. Caution should be taken in interpreting the model.

## Conclusion

Optimal control application was derived and analyzed to pattern the progression of COVID-19 cases in Indonesia. The SEI2RS model is a stable model, based on equilibrium results. Estimating the number of cases and the time period between control measure initiation and the peak number of cases is important for policymakers and public health officials to assess effectiveness of control measures in this exponential phase of the COVID-19 outbreak. According to this study, the Indonesian government should be prepared for an increasing number of cases in the middle of May 2020. Even after reaching the peak, the declining slope of infectious cases will be slow and even at the end of 2020, there will still be about 20,000 active cases.

It seems that COVID-19 will remain endemic in parts of the world, like other infectious diseases. The model provides evidence that while the control profile of Scenario 2 is implemented through the year, a number of COVID-19 cases will still exist in the end of 2020. Thus, if all of the control profiles were to be lost, the number of cases would presumably be higher than previously predicted.

## Data Availability

All the primary data and materials involved in this paper are from the published articles and web links, and they are all available online. If readers need more information about data and materials, please contact the corresponding author for data requests.

## Appendix 1

### Characteristics of equilibrium

The equilibrium point is formed by $\frac{dS}{dt}=\frac{dE}{dt}=\frac{d{I}_1}{dt}=\frac{d{I}_2}{dt}=\frac{dR}{dt}=0$.

7 $$ {\displaystyle \begin{array}{l}\varLambda \mathrm{N}+{\varphi}_2R+{\beta}_1E+{\theta}_2{I}_2-\frac{\alpha_1S{I}_1}{N}-\frac{\alpha_2S{I}_2}{N}-\frac{\alpha_3S{I}_2}{N}-\mu S=0\\ {}\frac{\alpha_3S{I}_2}{N}-{\beta}_1E-{\beta}_2E-\mu E=0\\ {}\frac{\alpha_1S{I}_1}{N}-{\gamma}_1{I}_1-{\gamma}_2{I}_1-{\mu I}_1=0\\ {}\frac{\alpha_2S{I}_2}{N}+{\beta}_2E+{\gamma}_2{I}_1+{\varphi}_1R-{\theta}_1{I}_2-{\theta}_2{I}_2-{\mu}_1{I}_2=0\\ {}{\gamma}_1{I}_1+{\theta}_1{I}_2-{\varphi}_1R-{\varphi}_2R-\mu R=0\end{array}} $$

The equilibrium point is obtained as follows.

8 $$ {\displaystyle \begin{array}{l}{S}^{\ast }=\frac{\varLambda \mathrm{N}+{\varphi}_2R+{\beta}_1E+{\theta}_2{I}_2}{\frac{\alpha_1{I}_1}{N}+\frac{\alpha_2{I}_2}{N}+\frac{\alpha_3{I}_2}{N}+\mu}\\ {}{E}^{\ast }=\frac{\alpha_3S{I}_2}{N\left({\beta}_1+{\beta}_2+\mu \right)}\\ {}{I}_1^{\ast }=0\\ {}{I}_2^{\ast }=\frac{\beta_2E+{\gamma}_2{I}_1+{\varphi}_1R}{\theta_1+{\theta}_2+{\mu}_1-\frac{\alpha_2S}{N}}\\ {}{R}^{\ast }=\frac{\gamma_1{I}_1+{\theta}_1{I}_2}{\varphi_1+{\varphi}_2+\mu}\end{array}} $$

The stability at the equilibrium point (8) is obtained by assuming *f*_1_, *f*_2_, *f*_3_, *f*_4_, and *f*_5_ in which $\dot{S},\dot{E},{\dot{I}}_1,{\dot{I}}_2$ and $\dot{R}$, consecutive. Then the linearization of system (7) can be written with the following Jacobian matrix [33]:

9 $$ {\displaystyle \begin{array}{c}J=\left[\begin{array}{cc}\begin{array}{ccc}\frac{\partial {f}_1}{\partial S}& \frac{\partial {f}_2}{\partial S}& \frac{\partial {f}_3}{\partial S}\\ {}\frac{\partial {f}_1}{\partial E}& \frac{\partial {f}_2}{\partial E}& \frac{\partial {f}_3}{\partial E}\end{array}& \begin{array}{cc}\frac{\partial {f}_4}{\partial S}& \frac{\partial {f}_5}{\partial S}\\ {}\frac{\partial {f}_4}{\partial E}& \frac{\partial {f}_5}{\partial E}\end{array}\\ {}\begin{array}{ccc}\frac{\partial {f}_1}{\partial {I}_1}& \frac{\partial {f}_2}{\partial {I}_1}& \frac{\partial {f}_3}{\partial {I}_1}\\ {}\frac{\partial {f}_1}{\partial {I}_2}& \frac{\partial {f}_2}{\partial {I}_2}& \frac{\partial {f}_3}{\partial {I}_2}\\ {}\frac{\partial {f}_1}{\partial R}& \frac{\partial {f}_2}{\partial R}& \frac{\partial {f}_3}{\partial R}\end{array}& \begin{array}{cc}\frac{\partial {f}_4}{\partial {I}_1}& \frac{\partial {f}_5}{\partial {I}_1}\\ {}\frac{\partial {f}_4}{\partial {I}_2}& \frac{\partial {f}_5}{\partial {I}_2}\\ {}\frac{\partial {f}_4}{\partial R}& \frac{\partial {f}_5}{\partial R}\end{array}\end{array}\right]\\ {}J=\left[\begin{array}{cc}\begin{array}{ccc}-\frac{\alpha_1{I}_1}{N}-\frac{\alpha_2{I}_2}{N}-\frac{\alpha_3{I}_2}{N}-\mu & \frac{\alpha_3{I}_2}{N}& \frac{\alpha_1{I}_1}{N}\\ {}{\beta}_1& -{\beta}_1-{\beta}_2-\mu & 0\end{array}& \begin{array}{cc}\frac{\alpha_2{I}_2}{N}& 0\\ {}{\beta}_2& 0\end{array}\\ {}\begin{array}{ccc}-\frac{\alpha_1S}{N}& 0& \frac{\alpha_1S}{N}-{\gamma}_1-{\gamma}_2-\mu \\ {}{\theta}_2-\frac{\alpha_2S}{N}-\frac{\alpha_3S}{N}& \frac{\alpha_3S}{N}& 0\\ {}{\varphi}_2& 0& 0\end{array}& \begin{array}{cc}{\gamma}_2& {\gamma}_1\\ {}\frac{\alpha_2S}{N}-{\theta}_1-{\theta}_2-{\mu}_1& {\theta}_1\\ {}{\varphi}_1& -{\varphi}_1-{\varphi}_2-\mu \end{array}\end{array}\right]\end{array}} $$

## Appendix 2

### Characteristics of optimal control

The necessary conditions for optimal control are satisfied with Pontryagin’s Minimum Principle.

10 $$ J\ \left({u}_1^{\ast },{u}_2^{\ast },{u}_3^{\ast },{u}_4^{\ast },{u}_5^{\ast}\right)={\displaystyle \begin{array}{c}\mathit{\min}\\ {}\Omega \end{array}}\ J\left({u}_1,{u}_2,{u}_3,{u}_4,{u}_5\right) $$

where Ω = {(*u*_1_, *u*_2_, *u*_3_, *u*_4_, *u*_5_)ϵ *L*^1^(April 10, 2020; December 31, 2020)| 0 ≤ *u*_*i*_ ≤ 1 for *i* = 1, 2, 3, 4, 5}.

Consider Lebesgue space *L*^*p*^(*ℝ*^n^). For *p* = 1 and *p* = 2, the space is composed of all functions *f* : *ℝ*^n^ → *ℝ* with

11 $$ \underset{{\mathbb{R}}^{\mathrm{n}}}{\int }{\left|f(x)\right|}^p dx<\infty $$

Normed space that formed is

12 $$ {\left\Vert f\right\Vert}_{p=1}:= \underset{{\mathbb{R}}^{\mathrm{n}}}{\int}\left|f(x)\right|\  dx $$

for *p* = 1 and

13 $$ {\left\Vert f\right\Vert}_{p=2}:= {\left(\underset{{\mathbb{R}}^{\mathrm{n}}}{\int }{\left|f(x)\right|}^2\  dx\right)}^{\frac{1}{2}} $$

for *p* = 2, and both of them fulfill three norm properties.

In case Ω = {(*u*_1_, *u*_2_, *u*_3_, *u*_4_, *u*_5_)ϵ *L*^1^(t_0_, t_f_)| 0 ≤ *u*_*i*_ ≤ 1 for *i* = 1, 2, 3, 4, 5}.

14 $$ {\left\Vert f\right\Vert}_{p=1}:= \underset{{\mathbb{R}}^{\mathrm{n}}}{\int}\left|f(u)\right|\  du $$

Property 1 clearly fulfill based on 0 ≤ *u*_*i*_ ≤ 1.

‖*u*‖ ≥ 0 for any *u* ∈ *U* = {*u*_1_, *u*_2_, *u*_3_, *u*_4_, *u*_5_}, and ‖*u*‖ = 0 if and only if *u* = 0.

Property 2 is shown as follows

‖*αu*‖ = |*α*| ‖*u*‖ for any *u* ∈ *U* and *α* ∈ *ℝ*.

Evidence conducted as discrete *αu*_1_ + *αu*_2_ + … + *αu*_*n*_ = *α* (*u*_1_ + *u*_2_ + … + *u*_*n*_).

Property 3 is shown as follows

‖*u* + *v*‖ ≤ ‖*u*‖ + ‖*v*‖, for any *u*, *v* ∈ *U*.

Evidence conducted as discrete (*u*_1_ + *v*_1_) + (*u*_2_ + *v*_2_) + … + (*u*_*n*_ + *v*_*n*_) = (*u*_1_ + *u*_2_ + … + *u*_*n*_) + (*v*_1_ + *v*_2_ + … + *v*_*n*_).

In case Ω = {(*u*_1_, *u*_2_, *u*_3_, *u*_4_, *u*_5_)ϵ *L*^2^(t_0_, t_f_)| 0 ≤ *u*_*i*_ ≤ 1 for *i* = 1, 2, 3, 4, 5}

Property 1

‖*u*‖ ≥ 0 for any *u* ∈ *U* = {*u*_1_, *u*_2_, *u*_3_, *u*_4_, *u*_5_}, and ‖*u*‖ = 0 if and only if *u* = 0. Based on 0 ≤ *u*_*i*_ ≤ 1, it represented as discrete ${\left({u}_1^2+{u}_2^2+\dots +{u}_n^2\right)}^{0.5}$ would be positive number and become 0 if and only if *u* = 0

Property 2 (cancelation proof)

‖*αu*‖ = |*α*| ‖*u*‖ for any *u* ∈ *U* and *α* ∈ *ℝ*. Cancelation conducted as discrete

$$ {\left(\alpha {u}_1^2+\alpha {u}_2^2+\dots +\alpha {u}_n^2\right)}^{0.5}\ \mathrm{as}\ \mathrm{not}\ \mathrm{equal}\ \mathrm{as}\ \alpha\ {\left({u}_1^2+{u}_2^2+\dots +{u}_n^2\right)}^{0.5} $$

By the normed space, *L*^1^ is used in this paper.

The necessary conditions can be generated from the Hamiltonian equation. Based on that principle, the system of Eqs. (4) and (5) is converted to the Hamiltonian (*H*) given by

15 $$ H=E+{I}_1+{I}_2+\frac{1}{2}{A}_1{u}_1^2+\frac{1}{2}{A}_2{u}_2^2+\frac{1}{2}{A}_3{u}_3^2+\frac{1}{2}{A}_3{u}_4^2+\frac{1}{2}{A}_3{u}_5^2+\sum \limits_{i=0}^5{\lambda}_i{g}_i $$

Where *g*_*i*_ symbolizes the right-hand side of the differential equation of the *i* th state variable. More notably, the state variable is analyzed. Where ${\dot{x}}_i$ is state equations containing $\dot{S},\dot{E},{\dot{I}}_1,{\dot{I}}_2,$ and $\dot{R}$. the state equation is obtained by ${\dot{x}}_i=\partial H/\partial {\lambda}_i.$

16 $$ {\displaystyle \begin{array}{l}\dot{S}=\varLambda \mathrm{N}+{\varphi}_2R+\left(1+{u}_2-{u}_5\right){\beta}_1E+\left(1+{u}_4\right){\theta}_2{I}_2-\left(1-{u}_1-{u}_5\right)\frac{\alpha_1S{I}_1}{N}-\left(1-{u}_1+{u}_3-{u}_5\right)\frac{\alpha_2S{I}_2}{N}-\left(1-{u}_1-{u}_5\right)\frac{\alpha_3S{I}_2}{N}-\mu S\\ {}\dot{E}=\left(1-{u}_1-{u}_5\right)\frac{\alpha_3S{I}_2}{N}-\left(1+{u}_2-{u}_5\right){\beta}_1E-\left(1+{u}_2-{u}_5\right){\beta}_2E-\mu E\\ {}{\dot{I}}_1=\left(1-{u}_1-{u}_5\right)\frac{\alpha_1S{I}_1}{N}-\left(1+{u}_1-{u}_5\right){\gamma}_1{I}_1-\left(1-{u}_1+{u}_3-{u}_5\right){\gamma}_2{I}_1-{\mu I}_1\\ {}{\dot{I}}_2=\left(1-{u}_1+{u}_3-{u}_5\right)\frac{\alpha_2S{I}_2}{N}+\left(1+{u}_2-{u}_5\right){\beta}_2E+\left(1-{u}_1+{u}_3-{u}_5\right){\gamma}_2{I}_1+{\varphi}_1R-\left(1+{u}_4\right){\theta}_1{I}_2-\left(1+{u}_4\right){\theta}_2{I}_2-{\mu}_1{I}_2\\ {}\dot{R}=\left(1+{u}_1-{u}_5\right){\gamma}_1{I}_1+\left(1+{u}_4\right){\theta}_1{I}_2-{\varphi}_1R-{\varphi}_2R-\mu R\end{array}}. $$

Furthermore, adjoint equations are ${\dot{\lambda}}_i=-\partial H/\partial {x}_i$ and follow transversality conditions ${\lambda}_i^{\ast}\left({t}_f\right)=0$. Where *i* = 1, …, 5. So,

17 $$ {\displaystyle \begin{array}{l}{\dot{\lambda}}_1=-\partial H/\partial S={\lambda}_1\left(\left(1-{u}_1-{u}_5\right)\frac{\alpha_1{I}_1}{N}+\left(1-{u}_1+{u}_3-{u}_5\right)\frac{\alpha_2{I}_2}{N}+\left(1-{u}_1-{u}_5\right)\frac{\alpha_3{I}_2}{N}+\mu \right)-{\lambda}_2\left(\left(1-{u}_1-{u}_5\right)\frac{\alpha_3{I}_2}{N}\right)-{\lambda}_3\left(\left(1-{u}_1-{u}_5\right)\frac{\alpha_1{I}_1}{N}\right)-{\lambda}_4\left(\left(1-{u}_1+{u}_3-{u}_5\right)\frac{\alpha_2{I}_2}{N}\right)\\ {}{\dot{\lambda}}_2=-\partial H/\partial E=-1-{\lambda}_1\left(1+{u}_2-{u}_5\right){\beta}_1+{\lambda}_2\left(\left(1+{u}_2-{u}_5\right){\beta}_1+\left(1+{u}_2-{u}_5\right){\beta}_2+\mu \right)-{\lambda}_4\left(1+{u}_2-{u}_5\right){\beta}_2\\ {}{\dot{\lambda}}_3=-\partial H/\partial {I}_1=-1+{\lambda}_1\left(1-{u}_1-{u}_5\right)\frac{\alpha_1S}{N}-{\lambda}_3\left(\left(1-{u}_1-{u}_5\right)\frac{\alpha_1S}{N}-\left(1+{u}_1-{u}_5\right){\gamma}_1-\left(1-{u}_1+{u}_3-{u}_5\right){\gamma}_2-\mu \right)-{\lambda}_4\left(1-{u}_1+{u}_3-{u}_5\right){\gamma}_2-{\lambda}_5\left(1+{u}_1-{u}_5\right){\gamma}_1\\ {}{\dot{\lambda}}_4=-\partial H/\partial {I}_2=-1-{\lambda}_1\left(\left(1+{u}_4\right){\theta}_2-\left(1-{u}_1+{u}_3-{u}_5\right)\frac{\alpha_2S}{N}-\left(1-{u}_1-{u}_5\right)\frac{\alpha_3S}{N}\right)-{\lambda}_2\left(1-{u}_1-{u}_5\right)\frac{\alpha_3S}{N}-{\lambda}_4\left(\left(1-{u}_1+{u}_3-{u}_5\right)\frac{\alpha_2S}{N}-\left(1+{u}_4\right){\theta}_1-\left(1+{u}_4\right){\theta}_2-{\mu}_1\right)-{\lambda}_5\left(1+{u}_4\right){\theta}_1\ \\ {}{\dot{\lambda}}_5=-\partial H/\partial R=-{\lambda}_1{\varphi}_2-{\lambda}_4{\varphi}_1+{\lambda}_5\left({\varphi}_1+{\varphi}_2+\mu \right)\end{array}} $$

The optimality conditions are obtained by *∂H*/*∂u*_*i*_ = 0, where *i* = 1, 2, 3, 4. So,

18 $$ {\displaystyle \begin{array}{l}{u}_1=-\frac{1}{A_1}\left[{\lambda}_1\left(\frac{\alpha_1S{I}_1}{N}+\frac{\alpha_2S{I}_2}{N}+\frac{\alpha_3S{I}_2}{N}\right)-{\lambda}_2\frac{\alpha_3S{I}_2}{N}+{\lambda}_3\left(-\frac{\alpha_1S{I}_1}{N}-{\gamma}_1{I}_1+{\gamma}_2{I}_1\right)+{\lambda}_4\left(-\frac{\alpha_2S{I}_2}{N}-{\gamma}_2{I}_1\right)+{\lambda}_5{\gamma}_1{I}_1\right]\\ {}{u}_2=-\frac{1}{A_2}\left[{\lambda}_1{\beta}_1E-{\lambda}_2\left({\beta}_1E+{\beta}_2E\right)+{\lambda}_4{\beta}_2E\right]\\ {}{u}_3=-\frac{1}{A_3}\left[-{\lambda}_1\frac{\alpha_2S{I}_2}{N}-{\lambda}_3{\gamma}_2{I}_1+{\lambda}_4\left(\frac{\alpha_2S{I}_2}{N}+{\gamma}_2{I}_1\right)\right]\\ {}{u}_4=-\frac{1}{A_4}\left[{\lambda}_1{\theta}_2{I}_2-{\lambda}_4\left({\theta}_1{I}_2+{\theta}_2{I}_2\right)+{\lambda}_5{\theta}_1{I}_2\right]\\ {}{u}_5=-\frac{1}{A_5}\left[{\lambda}_1\left(-{\beta}_1E+\frac{\alpha_1S{I}_1}{N}+\frac{\alpha_2S{I}_2}{N}+\frac{\alpha_3S{I}_2}{N}\right)+{\lambda}_2\left(-\frac{\alpha_3S{I}_2}{N}+{\beta}_1E+{\beta}_2E\right)+{\lambda}_3\left(-\frac{\alpha_1S{I}_1}{N}+{\gamma}_1{I}_1+{\gamma}_2{I}_1\right)-{\lambda}_4\left(\frac{\alpha_2S{I}_2}{N}+{\beta}_2E+{\gamma}_2{I}_1\right)-{\lambda}_5{\gamma}_1{I}_1\right]\end{array}} $$

## Abbreviations

COVID-19: Coronavirus disease 2019
SEIR: Susceptible, exposed, infectious, recovered
SEI2RS: Susceptible, exposed, carrier, infectious, recovery, susceptible
PUM: Person under monitoring
PUS: Patient under supervision
PCR: Polymerase chain reaction
OCT: Optimal control theory
